# Circadian clock, carcinogenesis, chronochemotherapy connections

**DOI:** 10.1016/j.jbc.2021.101068

**Published:** 2021-08-08

**Authors:** Yanyan Yang, Laura A. Lindsey-Boltz, Courtney M. Vaughn, Christopher P. Selby, Xuemei Cao, Zhenxing Liu, David S. Hsu, Aziz Sancar

**Affiliations:** 1 Department of Biochemistry and Biophysics, University of North Carolina School of Medicine, Chapel Hill, North Carolina, USA; 2 Department of Medicine, Duke University Medical Center, Durham, North Carolina, USA

**Keywords:** cryptochrome, transcription–translation feedback loop, nucleotide excision repair, cisplatin, colorectal cancer, xenografts, XR-seq, CRC, colorectal cancer, CRY, cryptochrome, NTS, nontranscribed strand, PER, period, RPKM, reads per kilobase pair per million total reads, SCN, suprachiasmatic nucleus, TCR, transcription-coupled repair, TTFL, transcription–translation feedback loop, TS, transcribed strand, XR-seq, excision repair sequencing, ZT, zeitgeber

## Abstract

The circadian clock controls the expression of nearly 50% of protein coding genes in mice and most likely in humans as well. Therefore, disruption of the circadian clock is presumed to have serious pathological effects including cancer. However, epidemiological studies on individuals with circadian disruption because of night shift or rotating shift work have produced contradictory data not conducive to scientific consensus as to whether circadian disruption increases the incidence of breast, ovarian, prostate, or colorectal cancers. Similarly, genetically engineered mice with clock disruption do not exhibit spontaneous or radiation-induced cancers at higher incidence than wild-type controls. Because many cellular functions including the cell cycle and cell division are, at least in part, controlled by the molecular clock components (CLOCK, BMAL1, CRYs, PERs), it has also been expected that appropriate timing of chemotherapy may increase the efficacy of chemotherapeutic drugs and ameliorate their side effect. However, empirical attempts at chronochemotherapy have not produced beneficial outcomes. Using mice without and with human tumor xenografts, sites of DNA damage and repair following treatment with the anticancer drug cisplatin have been mapped genome-wide at single nucleotide resolution and as a function of circadian time. The data indicate that mechanism-based studies such as these may provide information necessary for devising rational chronochemotherapy regimens.

Circadian rhythms are the intrinsic oscillations of ∼24 h period in physiological and behavioral functions (1). The fact that they are found in organisms ranging from cyanobacteria to humans and that they have evolved at least four times independently in nature is an indication that they confer a selective advantage (2). The molecular foundation of the mammalian circadian clock is a transcription–translation feedback loop (TTFL) (3, 4, 5, 6, 7, 8, 9, 10, 11, 12, 13, 14). In the TTFL, CLOCK and BMAL1 (or its paralog NPAS2) make the positive arm, and CRY (CRY1 and CRY2) and PER (PER1 and PER2) make the negative arm. The CLOCK-BMAL1 transcriptional activator and the CRY-PER transcriptional repressor generate the primary circadian loop with ∼24 h periodicity. This loop is consolidated by the secondary loop of NR1D1/2 and ROR nuclear receptors and further fine-tuned by kinases CK1δ/ε (15, 16, 17, 18, 19, 20) and ubiquitin ligases that control the activity and stability of the clock proteins. This basic molecular system is present in the suprachiasmatic nucleus (SCN) in the anterior hypothalamus of mammals as well as in essentially all peripheral tissues. However, the SCN is the master circadian clock, which receives light signals from the eye through special fibers of the optic nerves and synchronizes the clocks in peripheral organs according to time of day through endocrine and neural signals (3, 4, 5, 9, 10, 21), as illustrated in Figure 1*A*.

**Figure 1:**
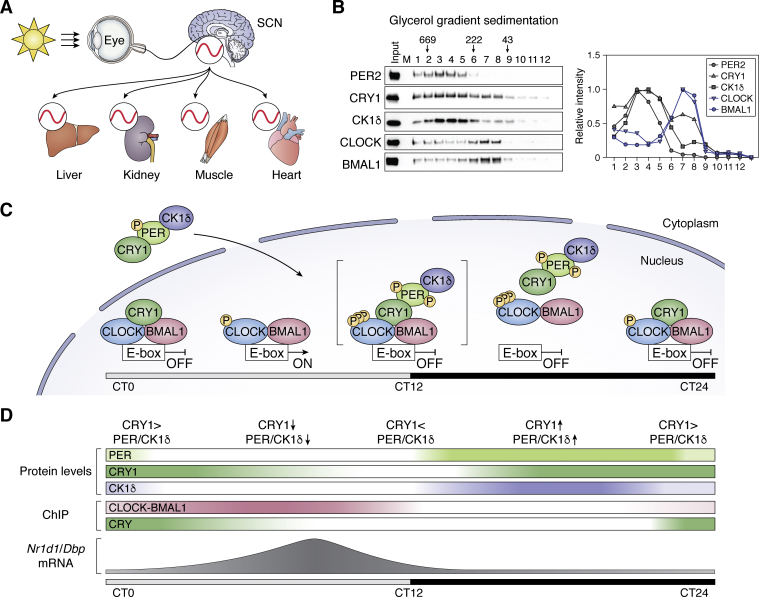
**Molecular mechanism of the mammalian circadian clock.***A*, model for circadian entrainment by light. The “master” clock in the suprachiasmatic nucleus (SCN) in the brain is entrained by neural input from photoreceptors in the retina. The master clock in turn maintains a coherent rhythmicity among clocks in peripheral tissue cells *via* neural signals and humoral factors. *B*, the positive (CLOCK-BMAL1) and negative (CRY-PER-CK1δ) arms of the TTFL are in two separate complexes. Mouse liver nuclei were harvested at ZT19 and the extract was separated by glycerol gradient velocity sedimentation along with reference proteins (thyroglobulin [669 kDa, 19S], β-amylase [222 kDa, 8.9S], and ovalbumin [43 kDa, 3.6S]). Fractions were probed by western blotting using appropriate antibodies. *Left panel*, western blot; *right panel*, quantitative scan of the western blot. CLOCK-BMAL1 sediments as a heterodimer (M_r_ ∼200 kDa), and PER2-CRY1- CK1δ sediments as a larger complex of M_r_ ∼500 kDa. *C*, TTFL model for the mammalian clock. The CLOCK–BMAL1 transcriptional activator binds to E-boxes at subjective dawn. At this time CRY1 is abundant and binds to the CLOCK-BMAL1-E-box complex and inhibits transcription (“Blocking type repression”). During the daytime, CRYs are degraded and CLOCK-BMAL1 activates transcription of target genes including *Cry* and *Per*. When CRY and PER accumulate, they enter the nucleus in the form of a CRY-PER-CK1δ complex, which transiently interacts with CLOCK-BMAL1-E-box (illustrated by *brackets*), phosphorylates CLOCK, and causes dissociation of the activator heterodimer (“Displacement type repression”). *D*, clock protein levels in mouse liver over the course of a circadian cycle. The levels are illustrated in the form of qualitative heatmaps, and the consequence of this clock protein change on clock-controlled *Nr1d1* and *Dbp* gene transcription over the course of the day is plotted. Adapted with permission from Cao *et al.* (25).

Although the basic mechanism of the mammalian clock is currently known, detailed mechanistic aspects at the molecular level, needed for possible medical intervention, are still being worked out. Figure 1 shows a model of the mammalian clock based on our recent work (22, 23, 24, 25) that incorporates earlier work by many investigators in the field. Figure 1*B* shows that the activating (CLOCK-BMAL1) and the repressive (CRY-PER-CK1δ) proteins are in two separate complexes. Figure 1*C* shows the relative abundance of various clock proteins with respect to their target sequence (E-box = CTGCAG) in DNA over the course of a daily cycle. The consequences of these protein–protein and protein–DNA interactions on transcription of clock genes and clock-controlled output genes in the mouse liver are shown in Figure 1*D*. At the beginning of the day (ZT = zeitgeber = 0), CLOCK-BMAL1 occupy their target E-box sequence, but cannot activate transcription because the repressor CRY is also abundant and binds to the CLOCK-BMAL1-E box complex, which prevents the transactivation domain of BMAL1 from interacting with transcriptional activators and thus inhibits transcription (“Blocking Type” inhibition). In the middle of the light phase, CRY levels are low and target genes are transcribed. In early evening (∼ZT12) PER accumulates, but in the absence of CRY, it cannot bind to CLOCK-BMAL1-Eboxes and cannot inhibit transcription. In the night phase, CRY and PER are abundant because of the uninhibited CLOCK-BMAL1-mediated transcription; CRY and PER enter the nucleus in the form of CRY-PER-CK1δ. Once in the nucleus, PER mediates phosphorylation of CLOCK by CK1δ and displacement of the entire complex from the promoter resulting in “Displacement Type” inhibition, which is followed by PER proteolysis and Blocking Type inhibition of CLOCK-BMAL1 target genes to reinitiate the cycle. Naturally this core mechanism is fine-tuned by kinases that affect all the clock protein activities and ubiquitin ligases that play roles in protein turnover (26). Having thus presented the core clock mechanism in its essential outlines, we now discuss studies that have investigated the effect of clock disruption on carcinogenesis and the attempts to use the circadian rhythm to improve the efficacy of chemotherapy.

## Circadian clock–carcinogenesis

Considering the overwhelming integration of the molecular circadian clock in gene expression and the fact that the circadian clock takes environmental cues (light, food) to synchronize gene expression, it would be expected that conditions that interfere with regular environmental or hormonal inputs would have serious pathological consequences, including metabolic syndrome, psychological problems, and cancer (9, 10, 11, 12, 13, 14, 27, 28, 29, 30, 31, 32, 33). In particular, clock disruption–carcinogenesis has been the focus of numerous studies as discussed below (27, 28, 29, 30, 31, 32, 33).

### Epidemiologic studies

**Figure 2:**
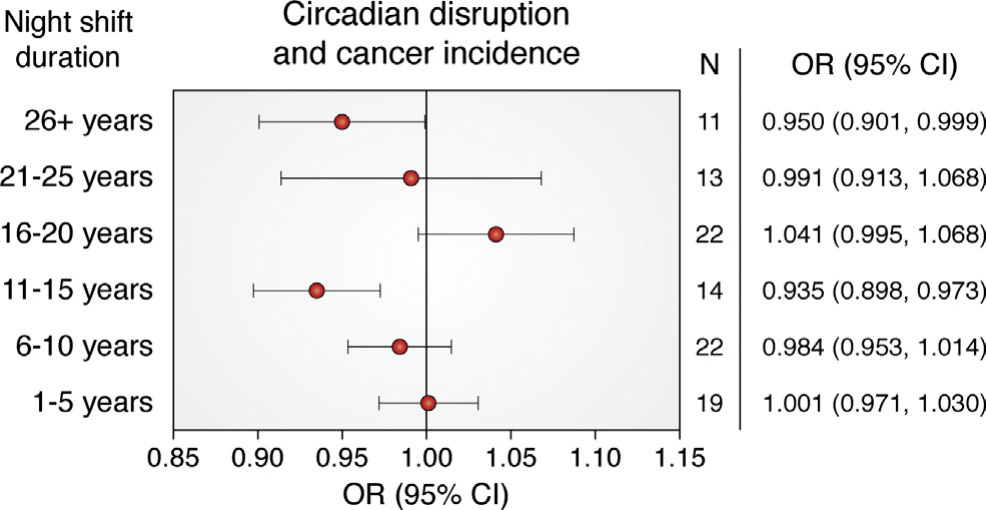
**Meta analysis studies on circadian disruption and cancer incidence.** The studies analyzed by Dun *et al.* (34) met the following criteria: (1) night-shift work was reported; (2) cancer risk was investigated; (3) cohort studies, case-control studies, or nested case-control studies; (4) the risk was estimated by odds ratio (OR), risk ratio, or hazard ratio, with 95% confidence interval (CI). Cancer risks among individuals with different classifications of night work duration (0–5, 6–10, 11–15, 16–20, 21–25, and ≥26 years) are plotted. Taking all eligible studies together, night-shift work did not increase the risk of cancer in any group of night workers. Image modified with permission from Ref.34 and used under Creative Commons.

For the past 25 years, numerous epidemiologic studies have been conducted to find out if there is a higher incidence of cancer in individuals with circadian clock disruption in the form of night-shift work by nurses or food industry workers, or chronic jetlag in flight attendants working transatlantic flights. The subject has been contentious with some epidemiologists concluding shift work is a carcinogen, whereas others claim otherwise. Nevertheless, the International Agency for Research on Cancer (IARC) in 2019 concluded that night-shift work was possibly carcinogenic [see (32)]. Interestingly, a paper published in 2020 that reported a systematic review of 57 observational studies with 8,477,849 participants (including the studies that were the basis of the WHO conclusion) “did not find an overall association between ever-exposure to night-shift work and the risk of breast, prostate, ovarian, pancreatic, colorectal, non-Hodgkin's lymphoma, and stomach cancers” (34) (Fig. 2). Thus, at present this debate is still ongoing (35, 36).

### Genetically modified animal model studies

**Figure 3:**
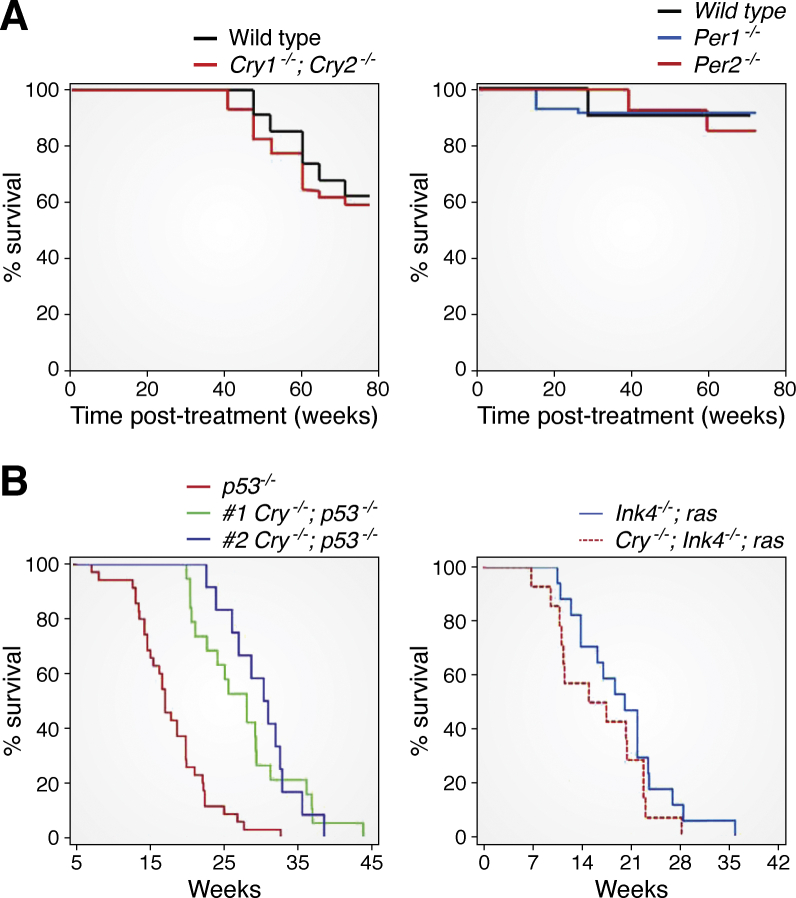
**Genetically modified model animal studies.***A*, Kaplan–Meier plots of death from cancer from two different studies of mice with clock gene mutations (31, 41). Eight-week-old mice of the indicated genotypes were exposed to 4 Gy of IR at ZT10 and observed for 80 weeks (*B*) Effect of *Cry* mutation on cancer incidence and mortality in mouse strains with a predisposition to cancer. Kaplan–Meier plots of death from cancer are shown. *Left*, *p53*^*−/−*^ (*red*) and *p53*^*−/−*^;*Cry1/2*^–/–^ (*green* and *blue*) survival probabilities. Data shown by the green line have been published (42), and the unpublished data shown by the blue line were obtained by a different member of the lab in a blind experimental design (31). (Right) Tumor-free survival of *ink4a*^*–/–*^*;ras(V12G)* (*blue*) and *ink4a*^*–/–*^*;ras(V12G);Cry1/2*^*–/–*^ (*red*) mice. The experiment was conducted in male mice maintained under standard conditions of 12 h light–12 h dark cycles and monitored regularly for the appearance of melanomas. There is no statistically significant difference between the two survival curves (*p* = 0.2), and hence, it is concluded that in this genetic background *Cry* mutation has no mitigating effect on cancer incidence or progression. Adapted with permission from Sancar *et al.* (31).

Considering the limitations of epidemiologic studies, once the mammalian core clock genes were identified, it seemed that the issue of clock disruption–carcinogenesis connection might be settled definitively by using mice with genetically modified (knockout) clock genes. *Clock* and *Bmal1* mutations did not predispose mice to cancer, but caused premature aging phenotypes (37, 38, 39). *Per1*^*−/−*^ or *Per2*^*−/−*^ mutations did not predispose mice to spontaneous and IR-induced cancers (40) (Fig. 3*A*). Similarly, *Cry1/2*^*−/−*^ mutant, which is arrhythmic under free-running conditions, is indistinguishable from wild-type with regard to spontaneous and IR-induced cancers (41). Interestingly, when the *Cry1/2*^*−/−*^ mutation is combined with *p53*^*−/−*^ mutation following a commonly used strategy to uncover the carcinogenicity of weakly penetrant tumorigenic genes, the opposite of the expected effect was found: the *p53*^*−/−*^ mice developed lymphomas and lymphosarcomas and had an average lifespan of 5.5 months, whereas the *p53*^*−/−*^*Cry1/2*^*−/−*^ mice developed tumors later and lived 1.5-fold longer than the *p53*^*−/−*^ mice (42). Thus, in this context *Cry* mutation plays an anticarcinogenic function (Fig. 3*B*). However, this is not a universal effect of *Cry* mutation: *Ink4a*^*−/−*^*,ras(V12G)* tumor suppressor/oncogene mutant mice develop melanomas with 100% incidence with light exposed areas, and the combination of *Ink4a*^*−/−*^*,ras(V12G),Cry1/2*^*−/−*^ did not affect melanoma incidence or survival (31), indicating that the antitumorigeneic effect of the *Cry* mutation is context-dependent.

Other experimental clock-disruption regimens in specific genetic backgrounds have been reported to have the opposite, procarcinogenic, effect. An early study, in which the clock was disrupted by suprachiasmatic nuclei (SCN) lesioning, found that Glasgow osteosarcoma and pancreatic adenocarcinoma xenografts grew faster in SCN-lesioned arrhythmic mice (4). Another study reported that breast cancer-prone *p53*^*R273OH/+*^ mice that were subjected to weekly LD inversion (chronic circadian rhythm disturbance) gained more weight and developed mammary tumors at a faster rate compared with controls (43). Yet, at the end of 30 weeks of circadian disruption, both the circadian disrupted and control mice had equal total incidence of mammary tumors. Moreover, since the circadian-disrupted group gained significantly more weight, it is unclear whether the early appearance of mammary tumors was due to weight gain, as it is known that being overweight is a risk factor for breast cancer in humans. Yet, another study reported that when mice with lung-specific *K-ras* and *p53* deletions were subjected to chronic jet lag, they exhibited higher tumor incidence and progression compared with controls (44). However, the effect was small, with the survival difference between the jet-lagged and control animals being just several days. In conclusion, clock researchers have used rather creative experimental designs to uncover the carcinogenic effect of clock disruption and have found very specific conditions for such an effect, but, in general, the carcinogenic effects have been small (45). Nevertheless, these experiments are valuable in terms of providing a foundation for more realistic conditions for testing the clock–cancer connection.

### Circadian clock–cell cycle connection

The circadian clock, like all other biochemical pathways/signaling networks, interfaces with genes/proteins that regulate the cell cycle. Specifically, the p21 and p27 proteins, which inhibit the G1/S transition kinase CDK4/6, and the Wee1 kinase, which phosphorylates and inhibits the G2/M transition kinases CyclinB1/CDKs, are controlled by the core circadian TTFL. In addition, TIMELESS, which is strictly a clock protein in the form of PER-TIM repressor in *Drosophila*, in mammalian cells functions in both the core clock by interacting with CRYs and participates in replication fork protection and the intra-S checkpoint by interacting with checkpoint kinases CHK1 and CHK2 (46, 47). Finally, some isoforms of the heat shock protein HSP90 exhibit a low-amplitude circadian pattern of expression and, through their effect on cell cycle progression, appear to mediate time-of-day-dependent efficacy of certain anticancer drugs (48). Therefore, it is to be expected that the circadian and cell cycles would reciprocally influence one another. This coupling of the two cycles was unambiguously demonstrated in an exhaustive experimental/computational study with circadian synchronized and proliferating mammalian cells in tissue culture (49). However, it was also pointed out that circadian cycle–cell cycle coupling is not essential for development and growth of animals with a genetically disrupted clock. Indeed, *Cry1/2*^*−/−*^ and *Per1/2*^*−/−*^ mice (and *Per^o^ Drosophila*) with no functional clock develop and grow normally (10, 31, 39). Apparently, the coupling of the two oscillators is not of such strength that its absence interferes with development. Nonetheless, both clockless mice and *Drosophila* exhibit reduced fecundity, which might be ascribed to the circadian effect on mating behavior (10).

### Circadian clock–oncogene/tumor suppressor connection

The major tumor suppressor gene *p53* and the two oncogenes mutated in most human cancers, *myc* and *ras*, have been mechanistically linked to the circadian clock.

#### p53

There is a complex relationship between p53 and the clock. CRYs have no effect on the life span of otherwise wild-type mice (7, 39). However, their absence extends the life span of *p53*^*−/−*^ transformed cancer cells prone to intrinsic and extrinsic apoptosis (50). Thus, while the clock does not affect *p53* expression directly, clock disruption by *Cry* mutation ameliorates the development of at least the progression of tumors caused by p53 mutation (Fig. 3*B*). In support of this antitumorigenic effect of CRY absence or downregulation, it was found that in low-risk and slow-progressing chronic lymphocytic leukemias, *Cry1* expression is silenced by aberrant CpG hypermethylation and that the methylation status of the *Cry1* promoter could be used as a prognostic marker (51, 52). In addition to these effects of CRY on p53 mutation-caused cancers, p53 also affects the molecular clock by regulating *Per2* transcription: p53 binds to a p53-response element in the *Per2* promoter, which overlaps with the E-box. As a consequence, *p53*^*−/−*^ mice have a short period and an overall unstable circadian rhythm (53, 54).

#### MYC

**Figure 4:**
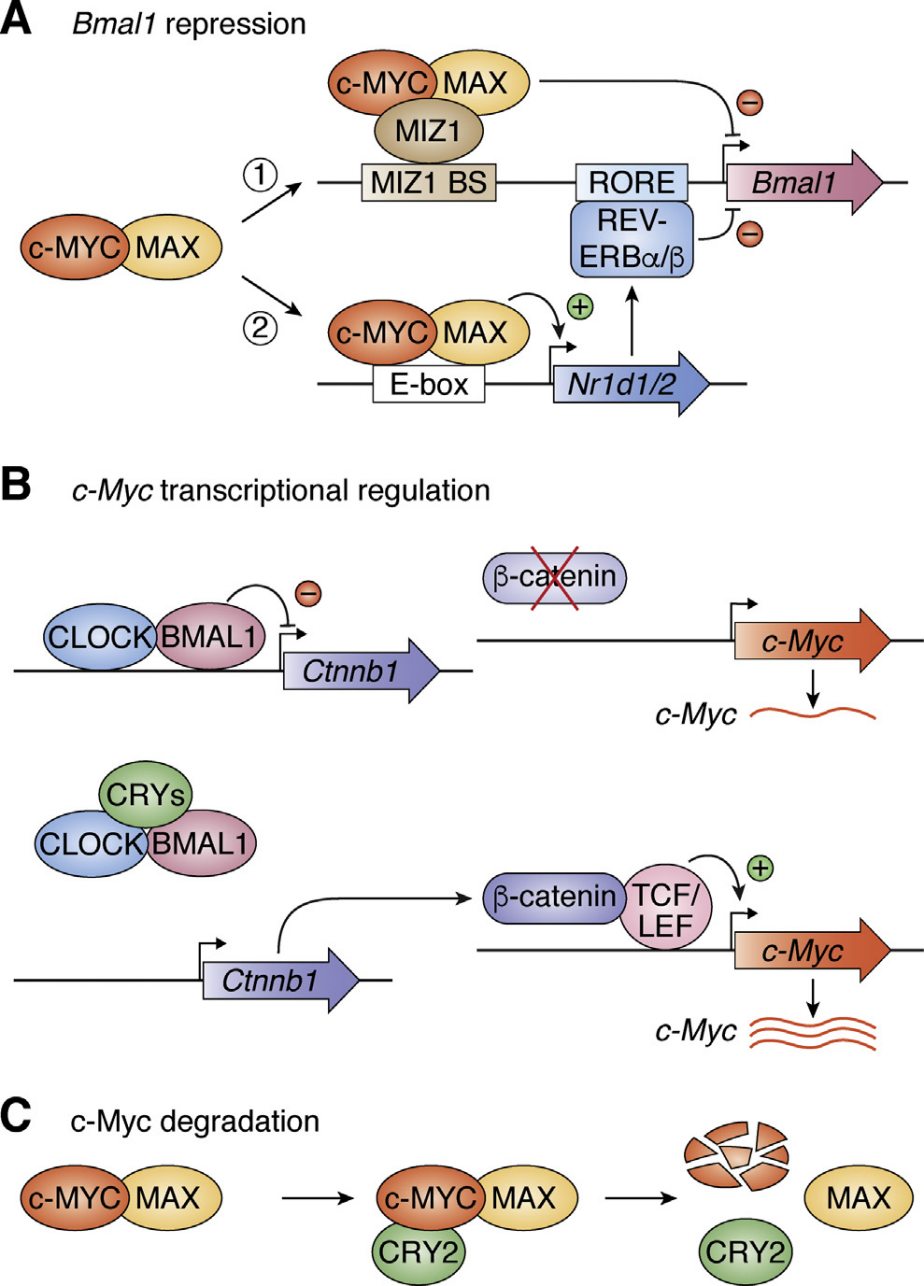
**Regulation of the clock by c-MYC and of c-MYC by the clock.***A*, c-MYC regulates *Bmal1* by two mechanisms (55, 56, 57, 58). First, c-MYC, in the form of c-MYC-MAX-MIZ1 heterotrimer directly binds to the MIZ-binding site upstream of the *Bmal1* promoter and directly inhibits its transcription. Second, in the form of c-MYC-MAX it binds to the E-boxes of the REV-ERB α/β genes (*Nr1d1/2*) and stimulates their transcription. NR1D1/2, in turn, binds to the RORE element of *BMal1* and inhibits its transcription. *B*, regulation of c-MYC at the transcriptional level by the clock (59). The β-Catenin gene (*Ctnnb1*) contains an E-box in its 35th intron, to which BMAL1-CLOCK bind and act as a context-dependent repressor (23, 24, 59) to interfere with the transcription of *Ctnnb1* (*top*). CRY-PER remove CLOCK-BMAL1 from the intron, activating *Ctnnb1* transcription (*bottom*). β-catenin makes a complex with TCF/LEF, which stimulates *c-Myc* transcription. In CRY mutants, BMAL1-CLOCK remains bound to the E-box of *Ctnnb1* intron and inhibits its transcription, and in the absence of, or with reduced levels of β-Catenin, *c-Myc* transcription is downregulated (*top*). *C*, regulation of MYC by the clock at a posttranscriptional level (60). When CRY2 is overexpressed by a strong promoter, such as the *Igu* promoter, it interacts with c-MYC and targets it for degradation by the ubiquitin/proteasome pathway, leading to reduced c-MYC levels.

c-MYC, like the proteins in the positive arm of the core clock, CLOCK/NPAS2-BMAL1, binds to E-boxes to regulate target genes. It affects the clock by multiple mechanisms (Fig. 4*A*) (55, 56, 57, 58). First, in the form of c-MYC-MAX-MIZ1, it binds to MIZ1-binding sites in the promoters/enhancers of the *Bmal1* and *Clock* genes and downregulates their expression, thus disrupting the clock. Second, the c-MYC heterodimer (or c-MYC-MAX) binds to the E-box in the *Nr1d1* promoter, upregulates its transcription, which, in turn, leads to downregulation of *Bmal1* by overproduced NR1D1/2, and ultimately disrupts the clock. Conversely, the clock appears to regulate c-MYC protein by transcriptional (59) and posttranscriptional (60) mechanisms (Fig. 4, *B* and *C*). In the transcriptional pathway, CLOCK-BMAL1 binds to the E-box in one of the introns of *β-catenin* and inhibits its transcription. β-CATENIN, in conjunction with TCF/TEF, is a transcriptional activator of *c-Myc.* Thus, inhibition of *β-catenin* expression by CLOCK-BMAL1 downregulates *c-Myc* expression. This inhibition is overcome by CRYs, which remove CLOCK-BMAL1 from the *β-catenin* intron. As a consequence, c-MYC expression is low in *Cry1/2*^*−/−*^ mice compared with WT mice (59). Thus, CRYs, which in general function as repressors, in this context function as activators, albeit indirectly (Fig. 4*B*). Secondly, it has been reported that CRY2 binds to phosphorylated c-MYC and targets its ubiquitylation and ultimate degradation by the proteasome (Fig. 4*C*). As a consequence, it was reported that in *Cry2* mutant mice, c-MYC was constitutively overexpressed and these mice had increased incidence of lymphosarcomas. However, this study used mice in which *c-Myc* was translocated to the *Eu(IgH)* locus (60), and therefore the two studies are not necessarily contradictory.

#### RAS

KRAS and the related members of the RAS family are mutated in nearly 50% of human cancers, and hence it would be expected that if RAS expression or function is modulated by circadian clock disruption, then clock gene mutations or chronic shift work would affect RAS signaling and its potential mutagenic effects. Two studies have addressed this issue (31, 61). In one, tumor-free survival of *Ink4a*^*−/−*^, *Ras(V12G)*, *Cry1/2*^*−/−*^ mice with no functional clock were used. These genetically engineered mice when maintained under LD12:12 developed melanomas with 100% penetrance and die within 30–35 weeks (31). Clock disruption by *Cry* knockout did not affect the tumorigenesis or the survival of the control *Ink4a*^*−/−*^*,Ras(V12G)* as determined by the conventional Kaplan–Meier plots (Fig. 3*B*). In the second study, from a circadian perspective, a rather intrusive study, *Kras* and *p53* were selectively deleted in mouse lungs, and then the mice were subjected to a chronic jet-lag regimen (61). Under these conditions, tumor incidence and the rate of progression increased. However, these effects were modest, with only a few days difference between the jet-lagged and control mice in both cancer incidence and progression rates. Finally, another study investigated the effect of RAS overexpression, and not surprisingly for a protein of such major signal transduction role in cell growth and differentiation, RAS overexpression in wild-type cells, as with p53 and MYC, leads to lengthening of the circadian period and senescent cell phenotype (61). Thus, on the whole, while as expected due to its global regulatory property, the circadian clock does intersect with signaling of oncogene and tumor suppressors with equally global cellular functions, these overlaps are not of sufficient magnitude for tight coupling of the clock with tumorigenesis or tumor suppression.

### Genomics of the clock–cancer connection

Several attempts have been made to correlate clock gene polymorphisms or expression levels in either cancer cell lines or tissues from various cancers to determine whether clock gene mutations (62) or levels of expression (63, 64, 65, 66, 67, 68, 69, 70) play a role in initiation or progression of cancer and susceptibility of cancer to a particular drug and a particular time of day for delivery of the drug (chronochemotherapy). The effects of clock gene polymorphism are small, and both predisposing and protective mutations were observed in comparable levels, and thus it is unclear whether these associations have a pathogenic role in the observed phenotypes (33). Similarly, the clock gene expression data lack a time dimension of sampling (71) and a mechanistic link between the observed clock gene expression changes and the tumorigenic pathways and the suggestion that the -omics findings have been supported by clinical chronochemotherapy trials (72, 73) is not in accord with the actual clinical trials. However, the clock gene expression profiles of a select number of cancers may have prognostic value (64, 65, 68).

## Circadian clock–chemotherapy (chronochemotherapy)

Chronotherapy is generally understood to mean administering anticancer drugs at certain times of the day as dictated by the circadian clock for maximum efficacy and minimal side effects (74, 75, 76). In fact, it has been shown that the toxic effects of endotoxin (77) and the anticancer drug cyclophosphamide (78, 79) exhibit a strong circadian pattern. With this general principle as a guide, a number of investigators have attempted chronochemotherapy for the past 50 years, long before the molecular mechanism of the clock was well understood. These efforts will be recapitulated below, but suffice it to state at the outset that chronochemotherapy is not routinely practiced in the United Sates and, possibly, any other country.

### Empirical clinical trials

An early clinical trial of chronochemotherapy of ovarian cancer with a small number of subjects reported a 4-fold increase in the 5-year disease-free survival with doxorubicin plus cisplatin chronotherapy compared with subjects receiving conventional drug administration (80, 81). A follow-up large multicenter study did not confirm this preliminary report, and currently, chronotherapy is not practiced by the American Gynecologic Oncology Group (82). Similarly, even though an anecdotal beneficial effect of chronotherapy has been reported in treatment of metastatic colorectal cancer (83, 84), a large multicenter study in the European Union did not show a beneficial effect for the entire patient cohort, but a minor beneficial effect for men and a larger harmful effect for women in terms of survival (85). To summarize, as of now there is no convincing clinical trial that chronotherapy of cancer is beneficial in the currently practiced form (32).

### Mechanism-based chronochemotherapy

Elucidation of the basic mechanism of the mammalian circadian clock at the molecular level has made it possible to attempt to develop chronochemotherapy regimens with some mechanistic foundation. Currently, two general approaches are used, tissue-culture-based methods and xenograft-based methods. The tissue-culture-based methods take advantage of the discovery that mammalian cells in tissue culture can be circadian synchronized by dexamethasone (49). Then, such synchronized cultures from various cancers are treated with anticancer drugs at different phases of the circadian cycle to identify the phase at which the cancer cells are most sensitive to the drug. In further elaborations of this approach, small-molecule inhibitors or stabilizers of various core clock proteins have been identified and tested in various versions of the cell-based circadian system to develop adjuvants in cancer treatment regimens (86, 87, 88, 89). However, it must be noted that analysis of synchronized cell cultures has shown that in this system essentially only the core TTFL clock genes and those in the secondary consolidating loop exhibit circadian rhythmicity, in contrast to hundreds to thousands of genes in most organs in mice, and presumably in humans, that exhibit circadian rhythmicity. For this reason, we have focused our research on developing chronochemotherapy regimens for cisplatin in mice.

**Figure 5:**
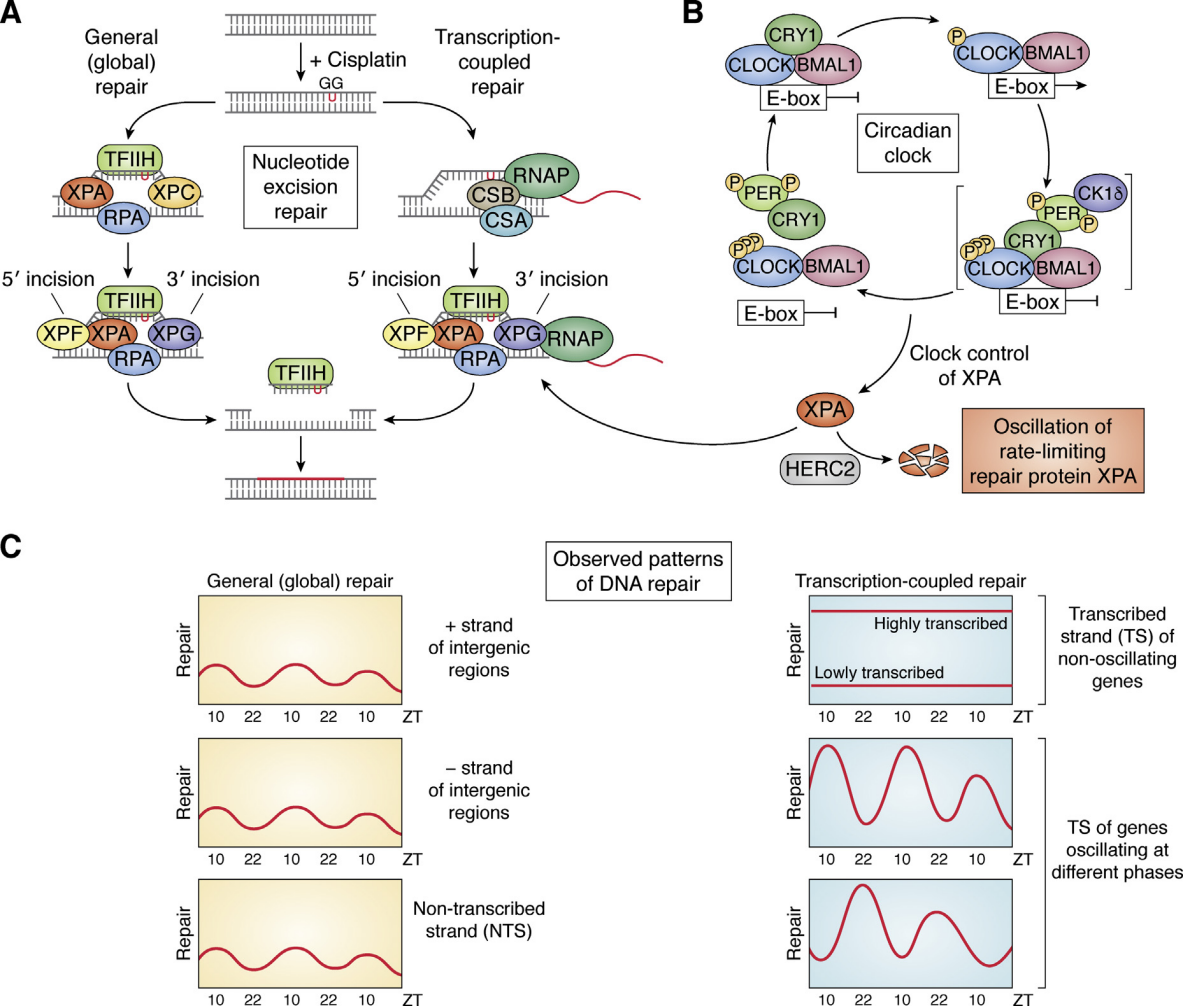
**Mechanism of nucleotide excision repair and its control by the circadian clock.***A*, molecular mechanism of mammalian Global and Transcription-Coupled Repair. Transcribed strand (TS) repair is predominantly determined by the phase of transcription of a given gene. The nontranscribed strand (NTS) repair is controlled by the repair enzyme complex oscillation, which is dictated by XPA damage recognition protein with a maximum at ZT10 for all genes regardless of the phase of transcription. *B*, schematic of the core clock that controls XPA expression. *C*, repair patterns of various gene's TS and NTS repair depending on whether they are constitutively expressed or controlled by the circadian clock, their phase of expression, and level of expression.

Cisplatin (and its second- and third-generation derivatives, carboplatin and oxaliplatin) is the most commonly used anticancer drug for treating cancers of solid tissues (90, 91). Cisplatin kills cells by making DNA diadducts, d(GpG) and d(ApG), and at much lower frequency interstrand cross-links. In humans and mice, nucleotide excision repair is the sole repair system for removing the major diadducts and thus preventing cell death (92). Excision repair in mammals is carried out by the concerted action of six repair factors (XPA, RPA, XPC, TFIIH, XPG, XPF-ERCC1), which make dual incisions ∼27 nucleotides apart bracketing the lesion. There are two pathways of excision repair (Fig. 5*A*), global repair and transcription-coupled repair (TCR) that differ in the damage recognition step (8, 93, 94). In global repair, XPC plays an essential role in the damage recognition step, whereas in TCR, RNA Polymerase II stalled at a lesion performs the damage recognition function, and as a consequence, the transcribed strand (TS) is repaired more efficiently (2–10-fold depending on the level of transcription) than the nontranscribed strand (NTS), and the global repair pathway repairs both strands in the regions of the genome that are not transcribed. For technical reasons, eukaryotic TCR cannot be performed with purified proteins or cell-free extracts (8), and at present, it can only be observed in tissue culture and living organisms (8, 92, 93, 95). Similarly, circadian control of global excision repair cannot be detected in tissue culture because of the limited circadian effect of synchronization procedures that only synchronize clock proteins and a limited number of clock-controlled genes (71). Thus, to detect the effect of the circadian clock on excision repair, we harvested mouse organs (liver, kidney, skin) over a circadian cycle and tested cell-free extracts made at each of these circadian time points for excision repair (21, 96, 97, 98). We found that in all tissues tested except testis, which is arrhythmic (99), repair was at a maximum at ZT10 and nadir at around ZT22 (Fig. 5*B*). When the expression profiles of the excision repair proteins were analyzed, it was found that transcription of the essential repair factor XPA exhibits circadian rhythmicity in these tissues coincident with the maxima of repair activity detected on Pt-d(GpG) substrates (96, 97, 100). However, since the *in vitro* system is not conducive to measuring TCR, we proceeded to test the combined actions of the global excision activity oscillation and circadian-controlled transcription on repair in mouse tissues. To this end, we developed a method called XR-seq (eXcision Repair-seq) to map repair throughout the whole genome at single nucleotide resolution (95, 101), and mapped cisplatin repair genome-wide in mice (Fig. 5*C*). First, we chose a single time point to measure repair in multiple mouse tissues (102), then measured repair over a circadian cycle (103), and finally measured repair in several sentinel genes over a clinically relevant time span (104). These studies have yielded information that will be potentially useful in improving the therapeutic index of cisplatin and in developing chronochemotherapy regimens and thus will be summarized below.

#### Multiple organ damage, repair, and transcription maps after cisplatin administration

**Figure 6:**
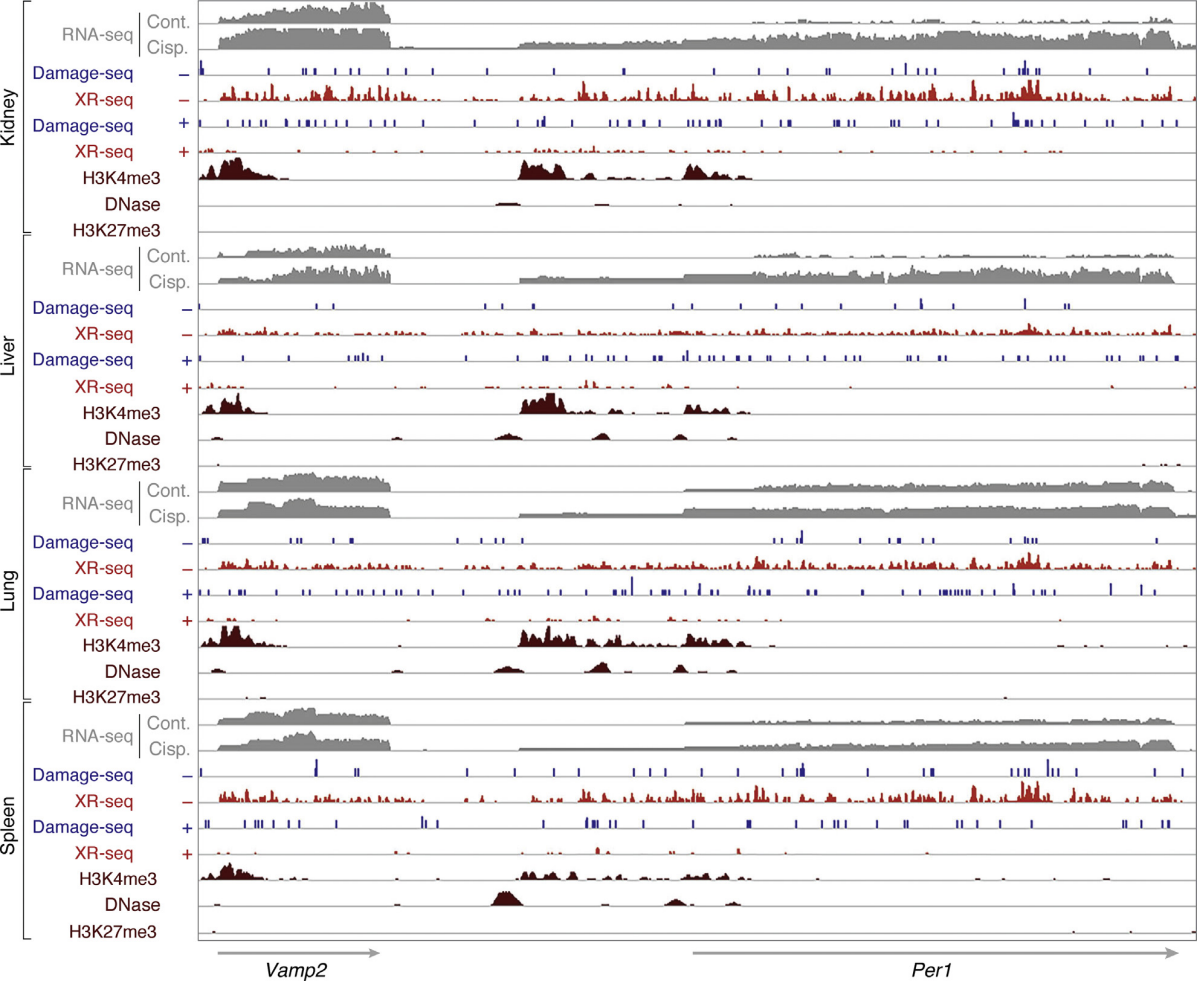
**DNA damage, repair, gene expression, and epigenomic markers for *Per1*.***Per1* is significantly upregulated after cisplatin treatment across all four organs. RNA-seq plus and minus cisplatin (cisp. and cont., respectively) is shown in gray at the top for each organ. Damage-seq and XR-seq data are shown for both strands. Pt-d(GpG) damage (Damage-seq) and repair (XR-seq) distribution on the TS and NTS are shown with − and +, respectively. Epigenetic data from ChIP-seq of H3K4me3 and H3K27me3, as well as DNase-seq, are plotted at the bottom of each organ. We show that the transcriptional and epigenomic profiles of *Per1* and neighboring regions across all four organs recapitulate the differences in DNA damage and repair between the TS and NTS. Adapted with permission from Yimit *et al.* (102).

Damage and repair maps were generated by Damage-seq and XR-seq, respectively; and transcription was quantified by RNA-seq (102). Cisplatin was administered by intraperitoneal (IP) injection and 4 h later, the liver, kidney, lung, and spleen were harvested and strand-specific DNA repair was analyzed at single-nucleotide resolution and compared with RNA-seq from the same tissues. Damage formation was the highest in the kidney, followed by the liver and lung, and the lowest in the spleen, in agreement with immunoslot blot data. XR-seq revealed the concordance of TS repair and RNA-seq, while the NTS was in general 5–10-fold less efficiently repaired compared with the TS in constitutively expressed genes and in genes in which the expression maximum phase coincided with the sampling time. As expected while some genes were in phase in all tissues, each tissue also exhibited rhythmic gene expression and a corresponding rhythmic repair pattern specific to that tissue. Perhaps one of the most significant findings of this study (Fig. 6) was the nearly 5-fold induction of *Per1* in all tissues after cisplatin administration as revealed by RNA-seq, and this increase in *Per1* transcription was accompanied by a similar level of increase of TS repair with only a minor effect on NTS repair, even though because of the 4 h delay between cisplatin administration and sampling, by this time the NTS contained more damage than the TS as revealed by Damage-seq. The significance of the *Per1* elevation on the circadian phase and amplitude remains to be investigated.

#### Effect of the circadian clock on cisplatin damage formation and repair

**Figure 7:**
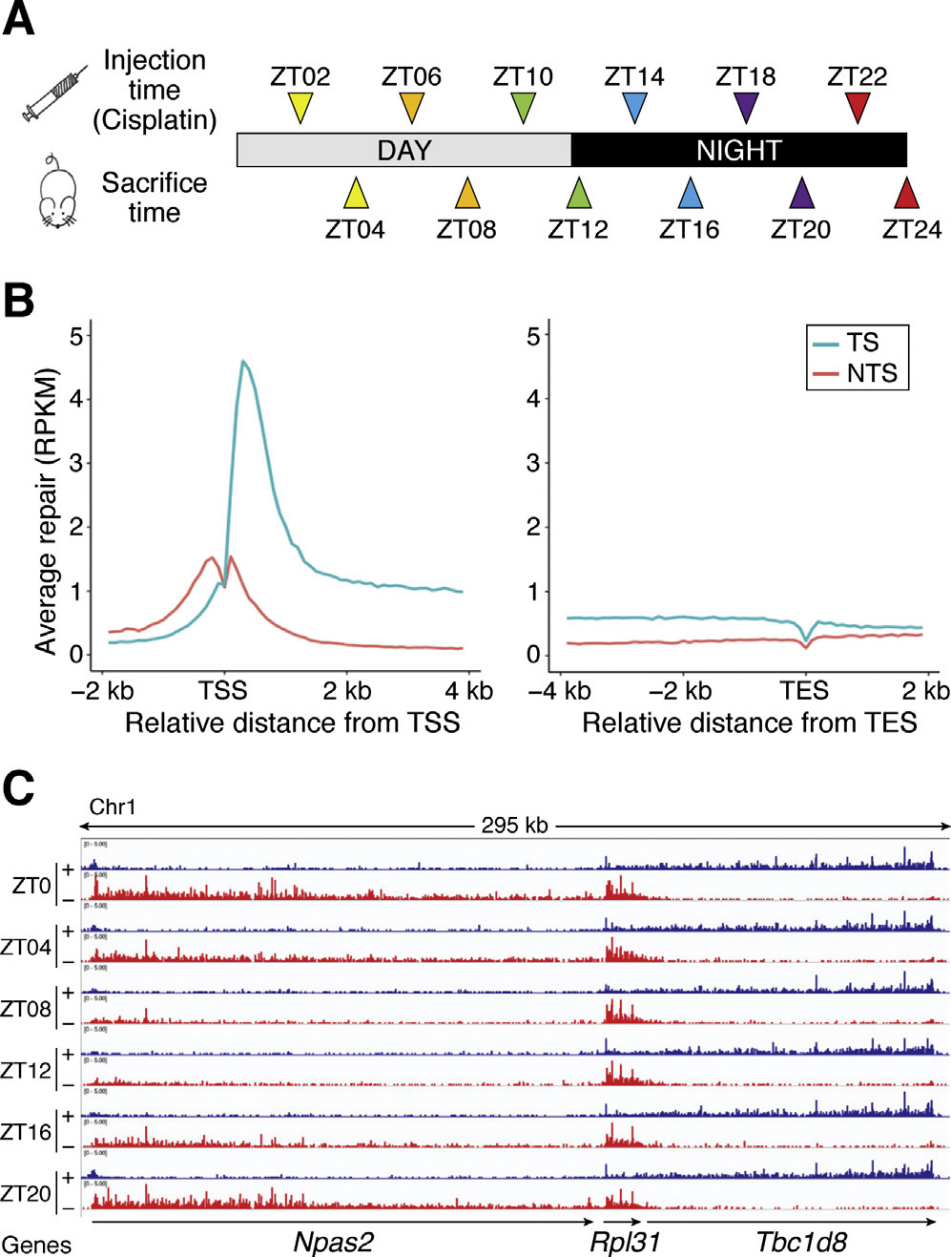
**Transcriptional and circadian control of excision repair of cisplatin-DNA adducts in mice.***A*, schematic of circadian repair experiment. Mice kept under 12-h light:12-h dark (LD 12:12) conditions were administered cisplatin at the indicated time points, and tissues were harvested 2 h later; the excision products were isolated from the liver and kidney and analyzed by XR-seq. ZT indicates circadian time where ZT0 is light-on and ZT12 is light-off. For each time point three mice were killed for XR-seq. *B*, genome-wide analysis of TS and NTS repair shows strong preference for TS repair in promoter-proximal regions, throughout gene bodies, and into the transcriptional end site (TES). Preferential repair reversal upstream of the transcription start sites (TSS) is due to bidirectional promoters for most mammalian genes such that the NTS in the gene body becomes the TS upstream of the TSS. The y axis shows reads per kilobase pair per million total reads (RPKM) for 100-nt windows. *C*, illustration showing the effect of transcription and the combined effects of the circadian clock and transcription on cisplatin repair. Repair patterns of a 295-kb region of chromosome 1 encompassing the *Npas2* clock gene and two neighboring genes are shown. *Blue*, plus strand XR-seq repair reads; *red*, minus strand XR-seq repair reads. The *Npas2* gene is itself clock-regulated and repair of its TS peaks at ZT20-ZT0 and troughs at ZT08. The clock output gene *Rpl31* exhibits much weaker rhythmicity in repair that is delayed compared with *Npas2* (peak ZT0-ZT08, minimum ZT16). *Tbc1d8* exhibits high amplitude and constant TS repair over the entire course of the circadian cycle. Adapted with permission from Yang *et al.* (103).

To establish a foundation for mechanism-based chronochemotherapy for cisplatin, we analyzed damage formation and repair of cisplatin administered to mice at 4 h intervals over a full circadian cycle (103) (Fig. 7*A*). Genome-wide repair at single nucleotide resolution revealed interesting features. First, genome-wide analysis of TS and NTS repair shows strong preference for TS repair at all time points and throughout the gene body of transcribed genes. The strand preference upstream of transcription start sites (TSS) is reversed because of the short promoter/enhancer transcript in the opposite direction (Fig. 7*B*). Interestingly, a screenshot of the repair pattern within a region of chromosome 1 that contains a strongly circadian-controlled gene (*Npas2*) and two adjacent genes that are weakly circadian (*Rpl31*) or noncircadian (*Tbc1d8*) and transcribed in opposite directions shows that the circadian-controlled *Npas2* gene exhibits strong circadian preference of TS/NTS repair, whereas *Rpl31* demonstrates weak rhythmicity and *Tbc1d8* maintains the same TS/NTS preference over the entire circadian cycle (Fig. 7*C*).

**Figure 8:**
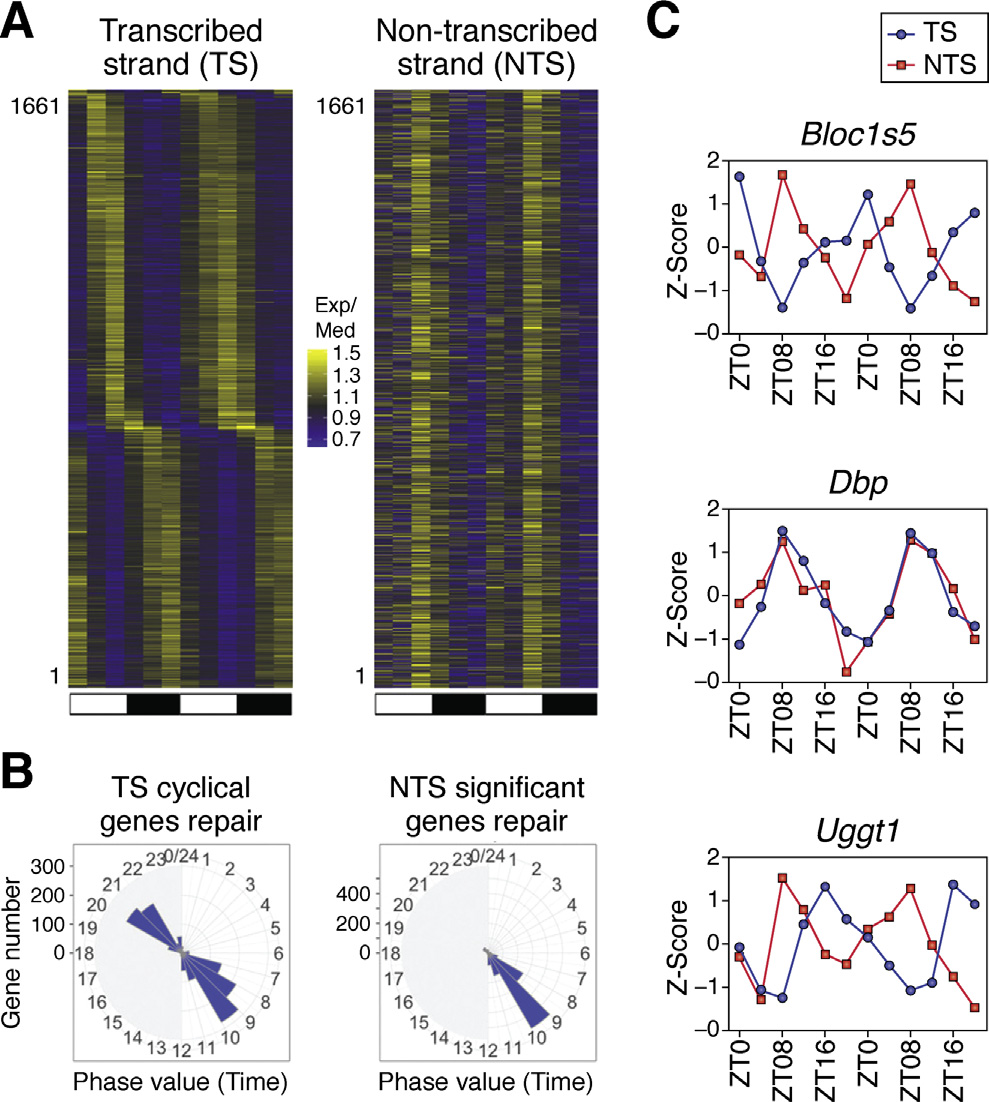
**Two interdependent circadian programs control repair of the TS and NTS.***A*, heatmaps of circadian repair cycles of the transcribed strand (TS) and nontranscribed strand (NTS) of 1661 highly rhythmic genes in mouse kidney. Exp/Med is, for each gene, RPKM at a given ZT divided by the median ZT RPKM value. Note the distribution of the repair maxima over the entire circadian cycle for the TS and the single maximum for repair of the NTS due to the circadian-controlled peak repair activity, which manifests itself on the NTS but its contribution to the TS repair is obscured by the much stronger effect of transcription on repair. The scale for selecting the significant cyclical genes is meta2d_pvalue <0.05, meta2d_rAMP >0.1. Each horizontal line represents one gene every 4 h from ZT0 to ZT24 with two replicates. *B*, radial diagram representation of TS and NTS repair. The TS repair exhibits two peaks corresponding to predawn and predusk, in agreement with numerous transcriptional analyses studies. The NTS repair exhibits a single peak at ZT08-11 in agreement with the peak transcription-independent excision repair activity. The scale for selecting the significant cyclical genes both in TS and NTS is meta2d_pvalue < 0.05, meta2d_rAMP > 0.1. *C*, examples of dissonance of the TS *versus* NTS repair. The dissonance is most apparent when the transcription/repair phase is farthest from ZT08, which represents the total repair activity and hence maximum NTS repair. We used three animals per time point for analysis and performed two biological replicates. The time range is from ZT0 to ZT24. In *C* (as in *A*), data for ZT0 to ZT24 replicate one are followed by data for ZT0 to ZT24, replicate two. Adapted with permission from Yang *et al.* (103).

With this background, we then analyzed the effect of the circadian clock on genome-wide repair of cisplatin damage in mouse kidney and liver. The repair heat map of 1661 genes that exhibit rhythmicity in TS repair with an amplitude of 2-fold in mouse kidney is shown in Figure 8*A*. The repair pattern exhibited rather interesting features. The TS of each gene controlled by the clock is repaired at the time of day dictated by the circadian clock-controlled transcription of that particular gene. This is because of the strong effect of transcription on TS repair (Fig. 5). In contrast, the NTS of all genes and both strands of nontranscribed genes and intergenic DNA are repaired maximally at one phase of the circadian clock (∼ZT10) (Fig. 8*B*). The circadian oscillation of the repair activity has negligible effects on TS repair because the effect of transcription on TS repair is of higher amplitude than the amplitude of the oscillation of the repair enzyme system. Finally, we note that these unique features of transcription and transcription enzyme circadian rhythmicities give rise to an interesting phenomenon at the single gene level: Depending on the circadian time of transcription, the TS and NTS of a particular gene might be repaired in-phase, in opposite phase, or in any of the phase relationships in between (Fig. 8*C*).

#### Effect of the clock and transcription on long-term repair kinetics of cisplatin damage

**Figure 9:**
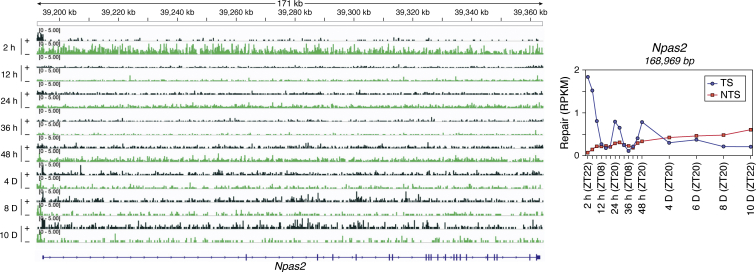
**XR-seq analysis of repair in circadian-controlled genes.** The screenshot shows repair profiles for the circadian-controlled gene*, Npas2*, which has a peak expression at ZT22. Repair of the rhythmic *Npas2* gene exhibits high amplitude transcribed strand (TS) (−s) repair peaks at 2 h, ∼24 h, and ∼48 h after drug injection. Only after 48 h does the nontranscribed strand (NTS) become the main source of repair product from the *Npas2* gene. RPKM reads per kilobase per million reads. Adapted with permission from Yang *et al.* (104).

Cisplatin causes cell death by inhibiting replication, interfering with transcription, and inducing apoptosis. To analyze the kinetics of cisplatin damage repair over a clinically relevant timespan, we followed gene and strand-specific repair of cisplatin damage in mouse liver over a 2-month period following cisplatin administration (104). We found (Fig. 9) that following cisplatin administration, the TS repair of a sentinel gene (Npas2) exhibited periodicity of ∼24 h, whereas the NTS repair proceeded at a slow and constant rate. The TS repair at its maximum was approximately 5-fold higher than the NTS and went down to a level lower than the NTS repair and went back up again in a circadian manner to the next peak ∼24 h later, whereas the NTS repair continued at the same slow rate. As a consequence of TCR, the TS repair was essentially complete within a week, while the NTS repair continued at its slow pace for 70 days, which was the duration of the experiment (100). Thus, in the clinical setting, these time-dependent differential rates of TS and NTS repair need to be considered in determining intervals of cisplatin dosing.

#### Human colorectal cancer xenografts as a model for chronochemotherapy

It has been reported that most cancers, at the tissue level, lack circadian rhythmicity, or if rhythmic, are out of phase with normal tissues (13). We wished to find out if this behavior can be used to advantage to develop more efficient chemotherapy regimens. We used colorectal cancer (CRC) xenografts to attempt to develop a chemotherapy regimen that minimizes side effects with maximum damage to cancer cells.

Xenografts from three CRC patients were grown in JAX NOD.CB17-Prkdc (SCID) mice. Once the tumor reached a size of 1 cm × 1 cm in diameter (500 mm^3^), six mice per patient xenograft were injected with cisplatin at 4 h intervals over a circadian cycle. Tumors were harvested 2 h after cisplatin injection, and Pt-d(GpG) repair in tumor tissue and liver and kidney of the host mouse was analyzed genome-wide by XR-seq. Results, to be further elaborated below, show that in general, circadian rhythmicity is lost in CRC xenografts (105). Whether the lack of rhythmicity in the tumor tissue is because of the inability of the population of cancer cells to maintain phase coherence or because of the disruption of the clock in individual cells as a consequence of carcinogenic transformation cannot be ascertained from our experimental system.

**Figure 10:**
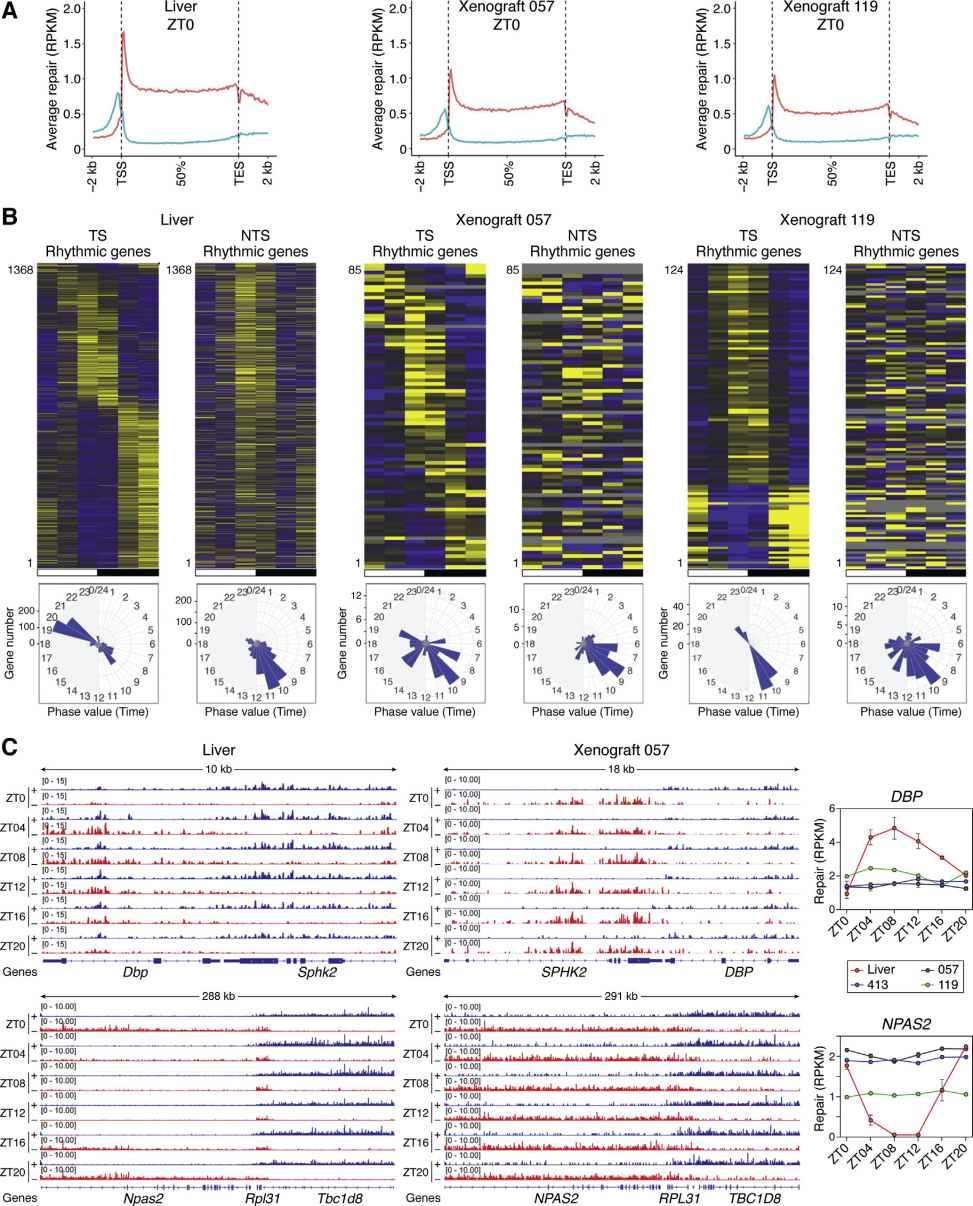
**Genome-wide analysis of TS and NTS repair in host liver and human colorectal cancer xenografts.***A*, plots of average TS and NTS repair across all genes in mouse liver and in cisplatin sensitive (057) or resistant (119) xenografts. XR-seq data obtained at ZT0 are plotted as RPKM average repair reads (*y* axis) along the length of a “unit gene” (*x* axis). The unit gene was constructed using all nonoverlapping human or mouse genes >5 kpb with a distance >5 kbp between adjacent genes. The unit gene is 100 bins in length, and values for average repair were obtained by dividing each gene into 100 bins and averaging the repair values for each successive bin for all genes from 1 to 100. Average repair 2 kbp upstream and downstream was similarly obtained. *B*, Heatmaps (above) and radial diagram representations (below) of circadian TS and NTS repair cycles in host liver and in cisplatin sensitive (057) and resistant (119) xenografts. In the heatmaps, each horizontal line (1368 lines for liver) represents repair of one gene from ZT0 to ZT20 at six time points. Exp/Med is, for each gene, RPKM at a given ZT time point divided by the median ZT RPKM value. The criteria for selecting the significant cyclical genes both in TS and NTS is meta2d_pvalue<0.05, meta2d_rAMP>0.1. Based on this scale, 1368, 85, and 124 genes were cyclical in host livers, xenograft 057, and xenograft 119, respectively. In an additional cisplatin resistant xenograft, 413 (not shown), 48 genes were cyclical. The host liver radial diagram (*left*) indicates two peaks of repair in the TS, predawn and predusk, and the NTS radial diagram to the right exhibits a single peak at ZT8-10 corresponding to our previous data (see Fig. 7). In xenografts, the TS and NTS repairs are less coherent but tend to exhibit a single peak at ZT8-11, likely due to the peak of global repair activity as described in Figure 8. *C*, repair of two representative circadian-controlled genes, *Dbp* and *Npas2*. The screenshots illustrate repair in liver (*left*) and in the cisplatin sensitive xenograft 057 (*middle*). It can be seen that in the liver, repair of the circadian-controlled genes (*Dbp*, *Npas2*) follows their transcriptional oscillation, while the respective neighboring genes, *Sphk*, *Rpl31*, and *Tbc1d8*, show constant repair over the entire circadian cycle. The graphs to the right illustrate quantitative values for TS repair as a function of circadian time for the liver, and for one sensitive (057) and two resistant (119, 413) xenografts. In contrast to the liver, rhythmic repair of the circadian-controlled genes is absent in the xenografts. From Sancar (105).

Figure 10*A* shows the genome-wide analysis of repair of transcribed genes in the livers of the host mice along with repair in xenografts. As apparent from the figure, both the livers of host mice and the xenografts of cisplatin-sensitive and -resistant CRC xenografts perform TCR to the same extent. Thus, for all practical purposes, repair in normal mouse tissue and cisplatin-sensitive and -resistant CRCs are of comparable efficiency and, in these cases at least, diminished or augmented excision repair capacity is not the cause of cisplatin sensitivity or resistance.

Next, we analyzed repair in host and in xenografts as a function of circadian time in the form of heatmaps and Radial Diagram representations (Fig. 10*B*). Liver data exhibited features essentially identical to our previously published data and were similar in the three host liver groups with peaks at ZT8-10 and ZT19-21 for the TS and a single peak at ZT10-12 for NTS. In contrast to the host liver with 1368 cyclic genes, xenografts range from 48 to 124 cyclic genes. A wild-type-like predawn/predusk TS repair pattern is seen in at least two of the xenografts, and NTS repair is widely distributed over the entire circadian cycle in two of the xenografts, indicative of lack of circadian rhythmicity in the excision repair enzyme activity. We also note that the genes that exhibit a circadian pattern of TS repair are not shared by the host liver and possibly reflect feeding pattern-dictated transcription of relevant genes.

Next, we analyzed circadian clock-controlled genes to illustrate the status of the primary TTFL genes and the genes of the consolidating secondary loop to gain further insight into the status of the molecular clock in the xenografts. In Figure 10*C* we show screenshots of representative genes controlled by E-box (primary TTFL) and RER element (consolidating NR1D1/2 loop). Figure 10*C* top panels show that while the livers of the host mice exhibit circadian rhythmicity and TCR of an E-box-controlled gene (*Dbp*), the xenografts, while exhibiting high TCR, no longer exhibit circadian control of this gene in terms of total transcription as measured by RPKM. Figure 10*C* bottom panels also show striking circadian rhythmicity in NR1D1/2-controlled *Npas2* in the liver of the mouse. In contrast, the rhythmicity is lost in the xenografts, but the relative transcription rate over the entire circadian cycle is high in all three xenografts. Finally, *Arntl(Bmal1)*, which is also primarily controlled by the secondary loop, has also lost rhythmicity, and this core clock activator gene maintains a constantly low level of expression in the xenografts (not shown). It is interesting that of all clock-controlled genes analyzed, only *Npas2* is constitutively expressed at a high level over the entire circadian cycle in xenografts relative to the host liver, which exhibits the well-known circadian pattern. In all other tested genes, including *Arntl (Bmal1)*, which like *Npas2* is mainly controlled by NR1D1/2, and secondarily by DBP, the expression pattern is uniformly low and at the level of the minimum of the circadian-controlled genes in normal tissues. Explanation of this observation requires further research into the circadian clock in normal human tissues.

To summarize, using XR-seq we have discovered that CRC xenografts perform global and transcription-coupled repair, but lack circadian rhythmicity in repair whether the xenograft is from a cisplatin-resistant or cisplatin-sensitive tumor. At present, the number of xenografts is insufficient for making generalizations and designing chronochemotherapy regimens based on this limited data. However, XR-seq is a powerful method for comparing repair in four dimensions in cancer and normal tissues and has the potential of aiding development of mechanism-based chronochemotherapy.

## Conclusions/perspective

Although circadian rhythms have been studied in great detail at a phenomenological level for nearly a century, the mechanistic foundation of the clock has only been elucidated over the past 25 years. The recent rapid progress in the field has revealed the pervasiveness of clock control of 50–90% of genes in mammals and in other organisms ranging from cyanobacteria to fruit flies. Moreover, sleep disorders caused by mutations in clock genes have been identified. Similarly, the interfacing of the clock with the cell cycle and all of the major signal transduction pathways has been established. Against this background, it has come as a major surprise that clock disruption by shift work or by clock gene mutations has not been found to be a significant contributing factor in carcinogenesis. Similarly, most of the genes/proteins that are targets for anticancer drugs, and therefore the efficacy of anticancer drugs, would be expected to be circadian time-dependent; however, research so far has not shown this to be the case. Yet it should be noted that the fine details of the circadian clock are still being worked out, and there is hope that with more advanced understanding of the mechanism of the human molecular clock, this knowledge will aid in developing more efficient approaches for cancer prevention and treatment.

## Data availability

The raw data and alignment data have been deposited in the Gene Expression Omnibus under accession number GSE178585.

## Conflict of interest

The authors declare that they have no conflicts of interest with the contents of this article.
